# Comparative genome analyses of *Mycobacteroides immunogenum* reveals two potential novel subspecies

**DOI:** 10.1099/mgen.0.000495

**Published:** 2020-12-09

**Authors:** Siew Woh Choo, Shusruto Rishik, Wei Yee Wee

**Affiliations:** ^1^​ College of Science and Technology, Wenzhou-Kean University, 88 Daxue Rd, Ouhai District Wenzhou, Zhejiang CN 325027, PR China; ^2^​ School of Science, Monash University Malaysia, Jalan Lagoon Selatan, Subang Jaya, Bandar Sunway, Selangor, MY 46150, Malaysia

**Keywords:** bacterial defence genes, comparative genomics, dN/dS, genomic islands, *M. abscessus*, *M. immunogenum*, non-tuberculous mycobacteria, rapidly growing mycobacteria, subspecies, taxonomy, virulence

## Abstract

*
Mycobacteroides immunogenum* is an emerging opportunistic pathogen implicated in nosocomial infections. Comparative genome analyses may provide better insights into its genomic structure, functions and evolution. The present analysis showed that *M. immunogenum* has an open pan-genome. Approximately 36.8% of putative virulence genes were identified in the accessory regions of *M. immunogenum *. Phylogenetic analyses revealed two potential novel subspecies of *M. immunogenum *, supported by evidence from ANIb (average nucleotide identity using blast) and GGDC (Genome to Genome Distance Calculator) analyses. We identified 74 genomic islands (GIs) in Subspecies 1 and 23 GIs in Subspecies 2. All Subspecies 2-harboured GIs were not found in Subspecies 1, indicating that they might have been acquired by Subspecies 2 after their divergence. Subspecies 2 has more defence genes than Subspecies 1, suggesting that it might be more resistant to the insertion of foreign DNA and probably explaining why Subspecies 2 has fewer GIs. Positive selection analysis suggest that *M. immunogenum* has a lower selection pressure compared to non-pathogenic mycobacteria. Thirteen genes were positively selected and many were involved in virulence.

## Data Summary

All genomic sequences were accessed from NCBI’s publicly available GenBank database. Please refer to Table S1 (available in the online version of this article) for a full list of the genome sequences used and corresponding assembly accession numbers.

**Impact Statement**

The number of nosocomial infections from the genus *Mycobacteroides* is showing an upwards trend. In the USA there has been an increase of 322 % between 1994 and 2014. As a member of this genus, *Mycobacteroides immunogenum* is an emerging pathogen. The dearth of comparative genomic studies for this species in the current literature motivated us to explore the genomes available on NCBI’s database. We found that the *M. immunogenum* species has an open pan-genome and is composed of two previously unknown subspecies. The contribution of horizontal gene transfer to subspecies divergence was explored. Subspecies 2 was found to be resistant to horizontal gene transfer due to having more bacterial defence genes. Positively selected genes were also identified and many were involved in virulence. Our work looks at the comparative genomics of an infectious disease-causing bacterium and lays a foundation for future genomic, functional and evolutionary studies into the pathogenicity of *M. immunogenum *. It is most relevant to researchers looking to study, diagnose and treat infections resulting from this species.

## Introduction

Mycobacteria, previously classified as the genus *Mycobacterium *, have recently been reclassified into the family *Mycobacteriaceae* containing five genera based on the core protein average amino acid identity of ~150 mycobacterial species [1]. The former NTM (non-tuberculous mycobacteria) *abscessus-chelonae* clade, to which *Mycobacterium immunogenum* belonged, has been renamed as the genus *Mycobacteroides *, and thus we use the new name *Mycobacteroides immunogenum* in this paper. To avoid confusion, the other members of the family *Mycobacteriaceae* will be referred to as mycobacteria hereafter.

*M. immunogenum* is a non-tuberculosis, rapid growing mycobacterium (RGM) that can be isolated from both environmental and human sources [2]. Many NTM members are exceptionally hard to grow in distilled water, and are resistant to organomercurials, chlorine, formaldehyde and alkaline glutaraldehyde in commonly used disinfectants [3]. They pose problems in hospital settings where they are implicated in infections related to surgical instruments and water supplies [3]. *M. immunogenum* can cause skin lesions [4], hypersensitivity pneumonitis and chronic obstructive pulmonary disorder [5] in both immunocompromised and immunocompetent patients in nosocomial infections [6]. It is also present in environmental sources such as metal working fluid [7], drinking water distribution simulation systems [8], swimming pools and bovine milk [9]. It forms biofilms at a rate that is typical of other NTM, but slower than its closest relative *M. abscessus* [10], probably allowing it to thrive in various stressful environmental conditions.

*M. immunogenum* is a relatively rare species [11]. In contrast, NTM surveillance in the eastern regions of China has identified *M. abscesuss–M. immuogenum* to be the second most common type of NTM [12], although the true frequency of *M. immunogenum* remains unknown. However, the number of the reported nosocomial infections caused by NTM has increased significantly. For instance, the number of reports rose by 322 % for the genus *Mycobacteroides* in the USA between 1994 and 2014 [13].

NTM have varied antibiotic resistance profiles and the infections they cause often cannot be treated with monotherapy. Therefore, the best treatment for *M. immunogenum* remains unknown and treatment must follow an antibiotic susceptibility test on a tissue culture [4]. For example, *M. immunogenum* and other members of *Mycobacteroides* have been found to be vulnerable to macrolides, such as clarithromycin, resistance to which is coded for by the *erm* gene [14]. *M. abscessus* has different *erm* gene patterns based on its subspecies, and therefore they have different treatment outcomes [15]. Furthermore, the treatment of infections involving NTM requires additional antibiotics such as amikacin, cefoxitin, imipenem, tigecycline, fluoroquinolone, linezolid and clofazimine as step-down therapy [16]. The primary treatment can last as long as 6 months and the step-down therapy up to 4 months [17]. Sometimes, surgical intervention is required as antibiotic treatment outcome can be variable [18]. An exploration of *M. immunogenum *’s genomic diversity is likely to be clinically relevant. Understanding the genetic makeup of this species may lead to better classification of potential subspecies and identification of genotypic signatures associated with sensitivities to antibiotics, and improve the management of diseases caused by *M. immunogenum *. Furthermore, it may provide clues as to its diversity and the emergence of *M. immunogenum* as a pathogen.

Here we performed comparative genome analysis of *M *. *immunogenum* with the aim to provide insight into the evolution, structure and functions of this human pathogen.

## Methods

### Genome annotation

The genome sequences of seven *M. immunogenum* strains and 24 other mycobacterial species were downloaded from the NCBI’s database (Table S1). For consistency, all genome sequences were re-annotated using the RAST server [19]. To identify restriction enzyme and toxin–antitoxin systems, database searches were performed using blastp [20] against REBASE [21] and TADB [22], respectively. To identify whether two genes were in the same cluster, we used a coverage threshold of 0.5 and a positive identity threshold of 0.5. CRISPRCasFinder [23] was used to identify putative CRISPR arrays and Cas proteins.

### Phylogenetic analyses at the genus or species levels

Phylogenetic trees were reconstructed using the single gene, multiple gene or core genome SNP approaches. For the single gene approach, we used 16S rRNA gene sequences predicted using Barrnap (Bacterial ribosomal RNA predictor) [24]. For the multiple gene approach, we used seven commonly used housekeeping genes, namely 16S rRNA, *rpoB*, *recA*, *hsp60*, *EF-Tu* [25]*, gyrA* [26] and *gyrB* [27], in order to reconstruct a more robust tree,. The sequences of the seven genes were concatenated into a supersequence and used for reconstructing a phylogenetic tree. For the core genome SNP approach, all genome sequences were uploaded to the PanSeq (Pan-genome Sequence analysis) web server [28]. The server aligned these sequences and identified the core genome regions and SNPs located in these highly conserved regions among all genomes. The percentage identity cutoff was set to 50 % and the core genome threshold was set to be the same as the number of genomes used. For example, if the dataset contains 25 genomes, the threshold was set to 25. All other parameters were left at their default values. The core genome SNPs were retrieved and used to generate a phylogenetic tree. All sequences were aligned using the muscle algorithm in mega 10 (Molecular Evolutionary Genetics Analysis) [29]. A maximum-likelihood tree was created with 1000 bootstrap replicates and the Kimura two-parameter model [30] using mega 10.

### *In silico* DNA–DNA hybridization

To further confirm the identity of species, *in silico* DNA–DNA hybridization (DDH) was calculated for the set of genomes containing seven *M. immunogenum* and one *M. abscessus* using average nucleotide identity by blast (ANIb) in JSpecies [31]. A threshold of 95–96 % ANIb was used as the cut-off point for species as this corresponds to the 70 % cut-off point for species delineation in *in vitro* DDH. Strains having ANIb values between ~96 and~99 % in strain-to-strain pairwise comparisons were assigned subspecies status [32].

A follow-up analysis was performed using the Genome-to-Genome-Distance-Calculator (GGDC) to corroborate species and subspecies boundaries. GGDC correlates well with *in vitro* DDH and has the added benefit of having confidence intervals [33]. GGDC provides three formulas for the calculation of DDH, termed Formulas 1, 2 and 3. Formula 2 was preferred as it calculates the sum of all identities found in high scoring pairs (HSP) and divides it by the length of the HSP and is thus independent of the length of the genome. A cut-off value of 70 % for Formula 2 was used to define the species boundary and 79 % to define the subspecies boundary [33].

### Pan-genome analysis

Two pan-genome analyses were performed using PGAP (Pan Genome Analysis Pipeline) [34]: one using 25 mycobacteria (having one representative strain for each species) and the other using seven *M. immunogenum* strains. To cluster the RAST-annotated genes, we used the Gene Family method from PGAP. The method works by using blast to perform pairwise comparisons between protein-encoding gene sequences from all the species. Two genes were assigned to the same cluster if they had ≥50 % sequence identity and 50 % sequence coverage, as well as an e-value of 1e^−10^.

To determine whether a pan-genome is closed or open, the subsets of the available number of genomes were taken. The combinations of these genomes were then taken, according to the formula *n*!/(*i!* (*n* – *i*)!) for *i* ∈ (1, 2, 3 … *n*), where *n* is the number of genomes in the subset. For example, *i* was taken to be 3 for a set containing eight genomes, so 8!/(3! (8 – 3)!)=56 genome combinations were taken. For each combination, the number of pan-genome clusters (total number of gene clusters) was calculated and determined to be one data point. The number of core gene clusters (genes common to all the selected genomes) was also determined to be one data point. This was repeated for subsets of genomes from 1 to the total number of genomes and all the data points were plotted. The following equations were fitted for pan-genome and core genome data points respectively:

$$Y_{1}=A_{1}\cdot x^{B_{1}}+C_{1}$$

$$Y_{2}=A_{2}\cdot x^{B_{2}}+C_{2}$$

For Equation 2, the *y_1_* value represents the number of clusters in the pan-genome. The number of genomes that were used to take a combination is represented by *x* and *A*_1_, *B*_1_ and *C*_1_ are fitting parameters. By extrapolating the fitted equation, it was determined whether the pan-genome is open or closed.

For Equation 3, the *y*_2_ value represents the number of clusters in the core genome. The number of genomes that were used to take a combination is represented by *x* and *A*_2_, *B*_2_ and *C*_2_ are fitting parameters. By extrapolating the fitted equation, it was determined whether most of the core gene clusters have been described or whether more core gene clusters are likely to be described if more genomes are analysed.

### Identification of species- and subspecies-specific genes

*M. immunogenum *-specific genes were identified by comparing the set of the *M. immunogenum* core genes with the set of mycobacterial core genes. For subspecies-specific genes, gene clusters found in one subspecies but not in another were identified. The calculations were performed using an in-house Python script using the orthologous gene cluster table produced by PGAP.

### Genomic islands (GIs) and prophage analysis

All GIs were predicted using the IslandViewer web server [35]. RAST-annotated GenBank files were uploaded to IslandViewer, which used Mauve [36] to reorder contigs by comparing to a reference genome if the draft genomes are uploaded. The GenBank files containing the reordered sequences were downloaded for visualization. The genomic locations for the GIs were also downloaded in tabular format for visualization. Prophage sequences were identified using the online server PHAST [37]. The GenBank files, annotated and reordered using IslandViewer, were uploaded to the servers for prophage annotation.

### BRIG visualization

Circular visualization was performed with the blast Ring Image Generator (BRIG) [38]. BRIG uses blast to compare query genomes against a chosen reference genome and displays regions of similarity based on a provided threshold. The upper threshold was chosen to be 90 % and lower threshold 80 %. Similarity is denoted by opacity: the more opaque the colour, the higher the similarity. The GI regions, prophage regions and the subspecies-specific gene annotations were supplied in the form of tab-delimited files with start and stop positions denoted.

### Positive selection analysis

One-is-to-one orthologues were identified from orthologue clustering of coding DNA sequences from seven *M. immunogenum* strains and five non-pathogenic mycobacteria (* M. smegmatis* INHR1, *M. hassiacum* DSM 44199, *M. gilvum* Spyr1, *M. vaccae* 95051 and *M. vanbaalenii* PYR1) and aligned with PRANK [39]. A cladogram was reconstructed using the core genome SNPs for the selected mycobacteria, using *M. immunogenum* marked as the foreground and the non-pathogenic mycobacteria marked as the background.

Positive selection analysis was performed using the CODEML module of the PAML package [40]. The branch-site model was used as it utilizes both the branch and different sites and has greater statistical power than the site-based and branch-based models [41]. Two models were fitted. In the null model H_0_, all the branches and sites were constrained to ω≤1. In the alternative model H_1_, the constraints were relaxed so that ω could be >1 for the foreground branches. The background branches were still constrained to ω≤1. The likelihood values produced by alternative hypothesis H_1_ was compared to the null hypothesis H_0_ and a Likelihood Ratio Test (LRT) value was produced using the following equation:

$$LRT=2(logL_{H}-logL_{H0})$$

where log *L*_H_ is the likelihood value for an alternative hypothesis and log *L*_H0_ is the likelihood value for the null hypothesis.

The LRT value was compared to a Chi-square distribution with a degree of freedom of 1 to determine significance [42]. A *P*-value of 0.05 was selected to be significant. Multiple comparison correction was performed using the Benjamini–Hochberg method [43] with a false discovery rate of 0.25. For orthologues with a significant LRT value, the alternative hypothesis fits better than the null hypothesis and thus positive selection is supported.

## Results

The genomes of seven *M. immunogenum* strains had an average genome size of 5.5 Mb with an average GC content of 64.0 %, slightly lower than the genus average (Table S1). The average number of protein-coding genes was 5563. Among the *M. immunogenum* strains, strain CD116 had the smallest genome size of 5.27 Mb, and also the smallest number of protein-coding genes of 5199.

### Phylogenetic relationship of *M. immunogenum* to other mycobacteria

**Fig. 1.**
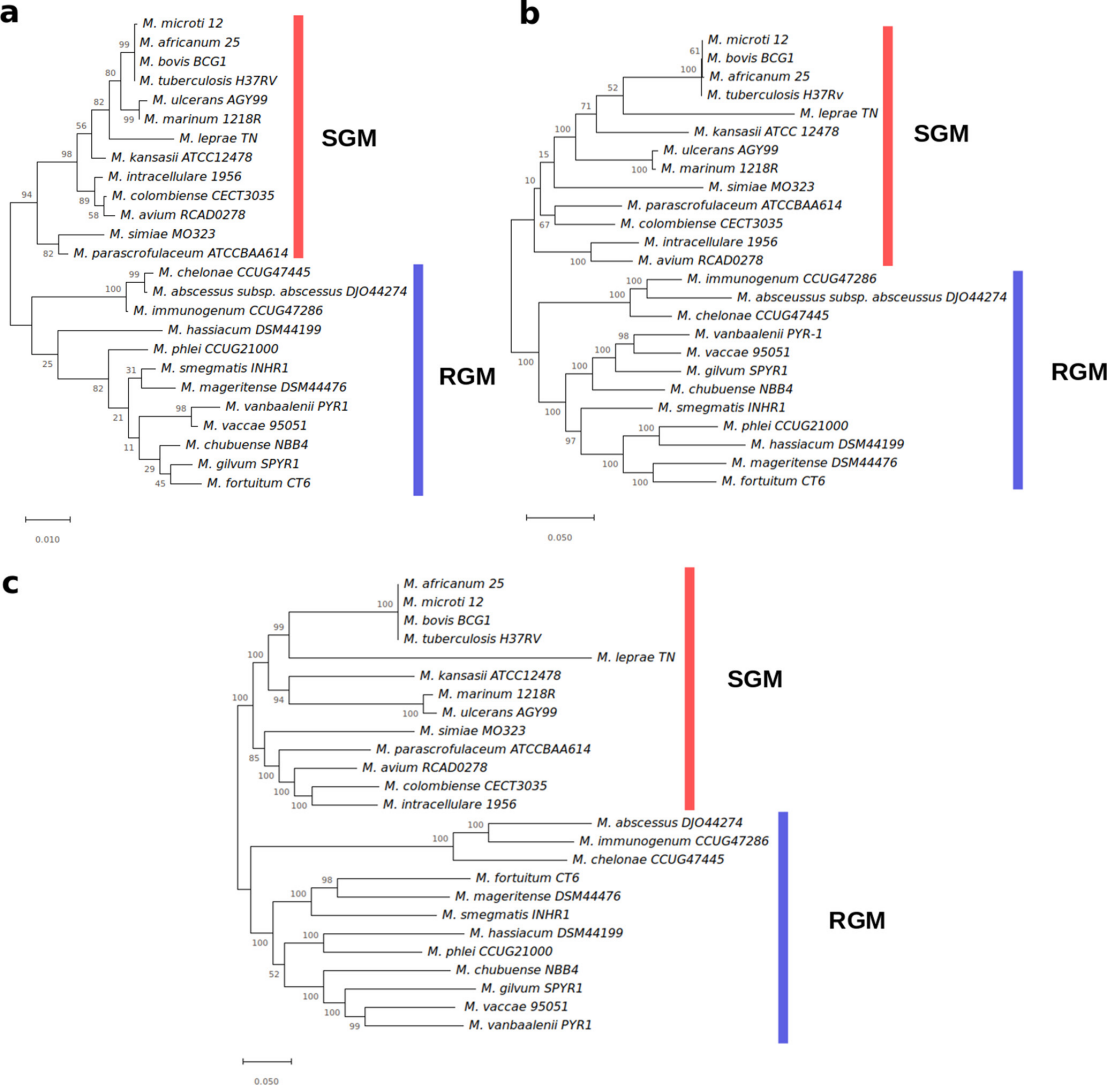
Maximum-likelihood phylogenetic trees of 25 representative mycobacterial species. (a) 16S rRNA sequence-based tree. (b) Multiple gene-based tree. (c) The core genome SNP-based tree. In total, 1000 bootstrap replicates were used. Orange and blue indicate the slow growing and rapidly growing mycobacteria, respectively. Branch length indicates divergence and bootstrap support values are also shown.

In our preliminary analysis, we reconstructed the 16S rRNA phylogenetic tree in order to gain better insight into the taxonomic positions of all *M. immunogenum* strains that we studied here (Fig. 1a). The 16S rRNA-based approach may not be sensitive and robust enough to discriminate mycobacterial species [44], and therefore we also performed phylogenetic analyses using multiple housekeeping genes (16S rRNA, *elon-Tu*, *gyrA*, *gyrB*, *hsp60*, *sup (Cu-Zn*), *recA* and *rpoB*] and also the core genome SNPs to cross-validate our findings (Fig. 1b, c). As expected, the slow growing mycobacteria (SGM) and RGM were clearly separated in all three trees that we reconstructed. *M. immunogenum* was classified as an RGM and this result was consistent in all trees. The closest relatives of *M. immunogenum* were *M. abscessus* and *M. chelonae *.

### Phylogenetic tree of *M. immunogenum*

**Fig. 2.**
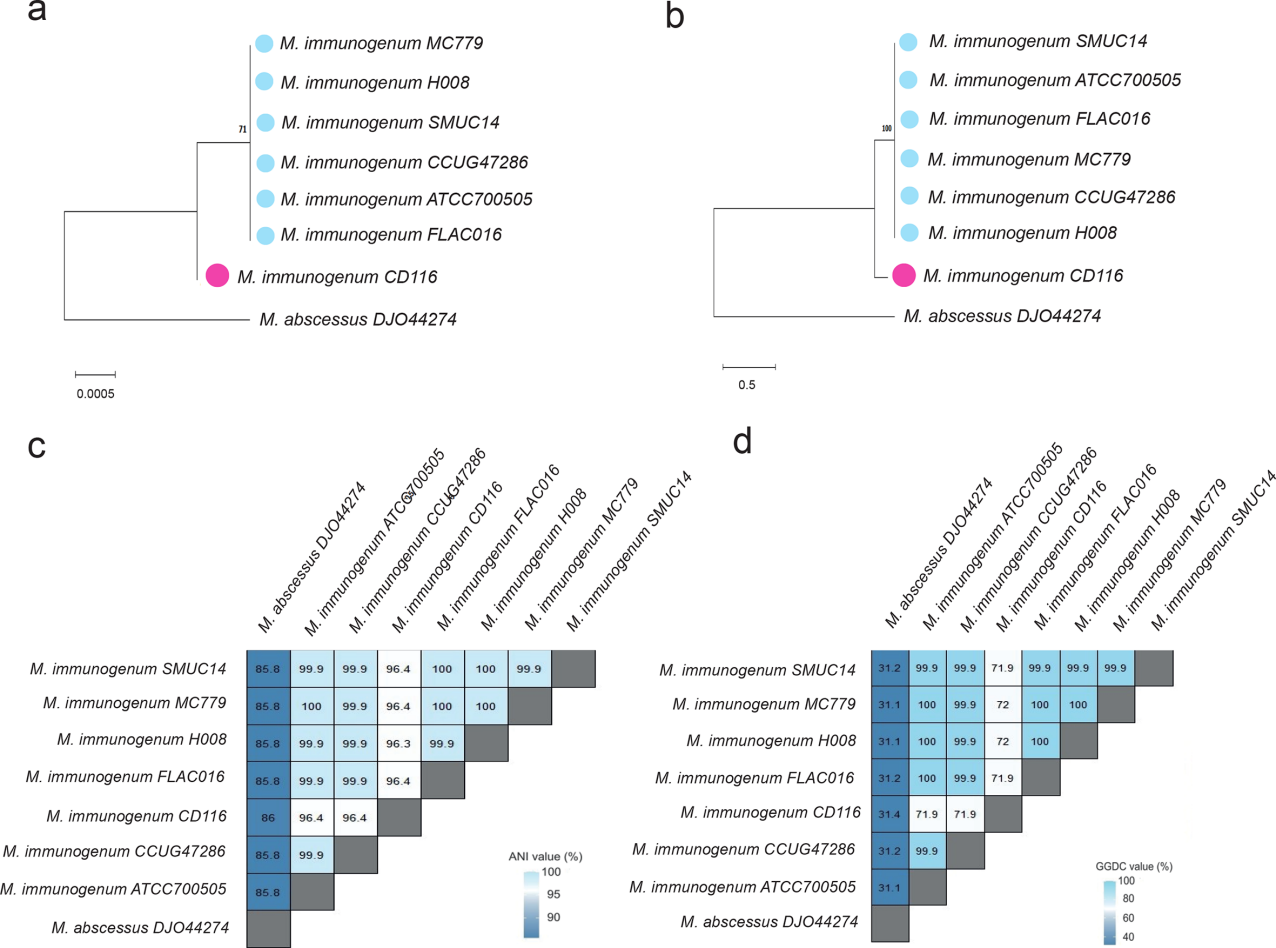
Maximum-likelihood phylogenetic trees based on 1000 bootstrap replicates created for seven *M *. *immunogenum* strains and *M. abscessus* as an outgroup. (a) 16S rRNA*-*based tree. (b) The core genome SNP-based tree. CD116 is found in a separate cluster from the other six *M. immunogenum* strains, which were in another cluster. Orange and blue indicate putative Subspecies 1 and Subspecies 2 respectively. Branch length indicates divergence and bootstrap support values are shown. (c) ANIb analysis. Genomes having an ANIb value of less than 96 % were defined as members of different species. Genomes with ANIb of 96–99 % were defined as the same species or subspecies. (d) GGDC analysis. GGDC values for the comparison between *M. immunogenum* CD116 (Cluster 2) with *M. immunogenum* strains (Cluster 1) and *M. abscessus* as an outgroup. Genomes with DDH values >70 % were defined as from the same species. Genomes with DDH values of 70–80 % were defined as members of the same species, but different subspecies.

After confirming the taxonomic position of *M. immunogenum* in the genus *Mycobacterium *, we reconstructed *M. immunogenum* trees using all seven *M. immunogenum* strains and *M. abscessus* as an outgroup in order to investigate their taxonomic relationships using the 16S gene and the core genome SNP approaches (Fig. 2a–b). Interestingly, the *M. immunogenum* strains grouped into two distinct clusters. Cluster 1 consisted of almost all *M. immunogenum* strains, whereas cluster 2 consisted of only one strain (CD116). This topology was consistent in all trees generated by three different approaches, suggesting that *M. immunogenum* may comprise two subspecies.

### ANIb analysis

To further confirm *M. immunogenum* comprises two subspecies*,* we performed ANIb analysis, which incorporates the ANI algorithm by Richter and Rossello-Mora [31]. The pairwise ANIb results between the genome sequences of *M. immunogenum* strains and *M. abscessus* were calculated (Fig. 2c). As anticipated, *M. abscessus* had lower pairwise ANIb similarity values (species cutoff of <96 %) with all the *M. immunogenum* strains*. M. immunogenum* CD116, on the other hand, had a pairwise ANIb similarity value of ~96.4 % with other *M. immunogenum* strains. Although CD116 had a lower ANIb value compared to the other *M. immunogenum* strains, it still had ANIb similarity values of 96–99 %, suggesting that it is still a member of *M. immunogenum* species, but probably represents a novel subspecies of *M. immunogenum *.

### DNA homology using GGDC analysis

As *M. immunogenum* strain CD116 had an incomplete genome, the results obtained may have been due to an incomplete sequence as it had a smaller genome size compared to the other *M. immunogenum* strains (5.27 Mb compared to 5.54–5.72 Mb). In order to corroborate ANIb results, *in silico* DDH of strain CD116 against the other seven genomes was also performed with the well-established GGDC method because this is independent of genome length and robust against incomplete genomes. The DDH method is a gold standard for differentiating species of the same genus. The widely accepted species boundary set by the DDH method is 70 %. The GGDC is an *in silico* method for traditional experimental DDH analysis that calculates the DDH of different strains. Analyses showed that strain CD116 (Cluster 2) and the members of Cluster 1 had DDH values between 71.9 and 72 % with a confidence interval of 68.90–74.80 % (Fig. 2d). Since the DDH values were within the range 70–80 %, it was suggested that strains CCUG47286, FLAC016, ATCC 700505, H008, MC779 and SMUC14 can be considered as representing putative Subspecies 1, whereas CD116 is considered as representing putative Subspecies 2.

### *M. immunogenum* has an open pan-genome

**Fig. 3.**
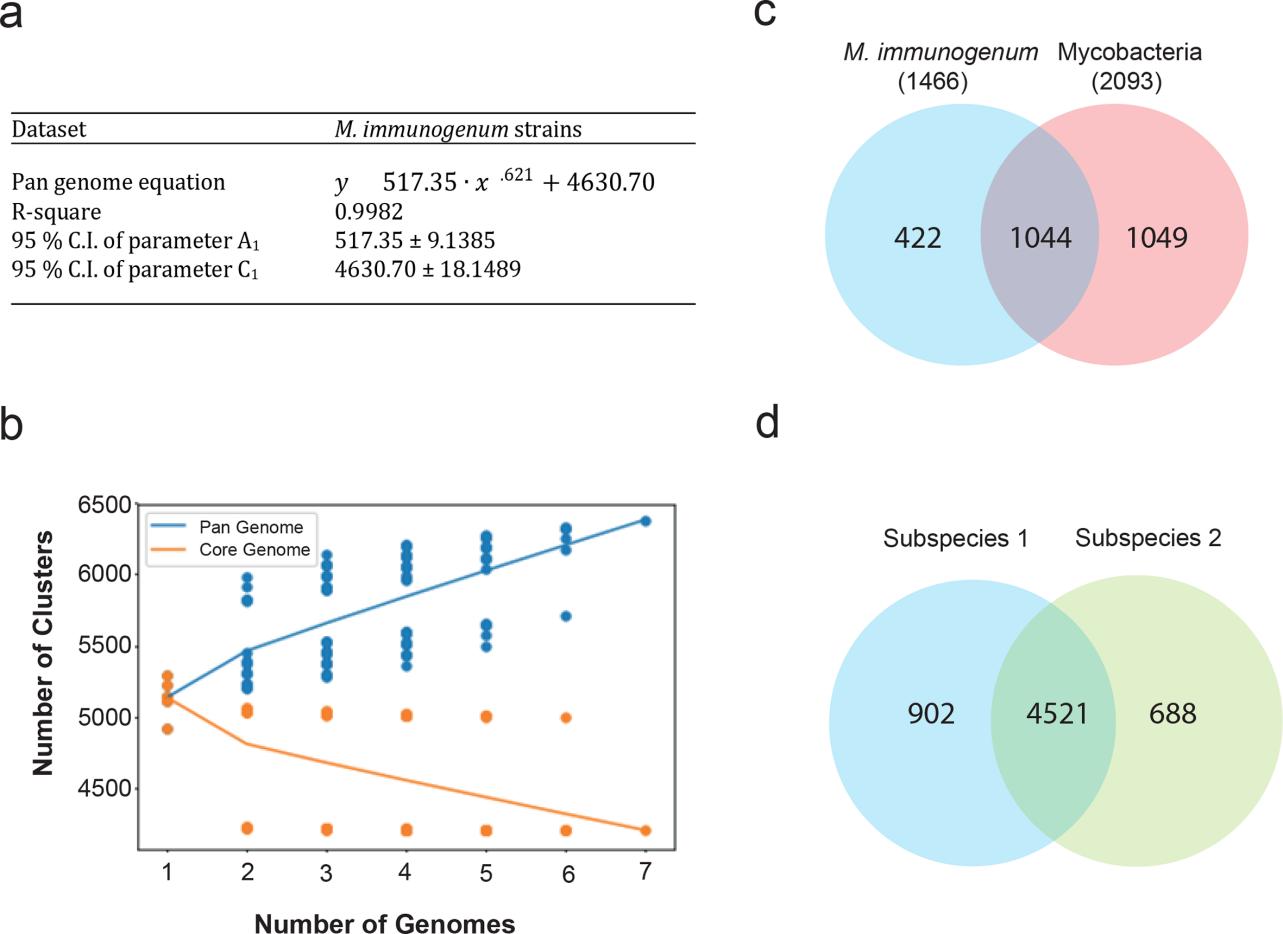
Pan-genome analysis. (a) Parameters obtained after fitting Equation 2 (Methods), $y_{1}=A_{1}\cdot x^{B_{1}}+C_{1}$, to the pan-genome cluster number vs. number of genomes for the corresponding data sets. (b) Plot of the number of gene clusters in the pan-genome and core genome vs. number of genomes sampled for seven *M. immunogenum* strains. The blue data points indicate the number of pan-genome clusters (total number of clusters) belonging to the sampled number of genomes. Likewise, the orange data points indicate the number of core genome clusters, i.e. gene clusters present in the sampled number of genomes. (c) Venn diagram showing the relationship between *M. immunogenum* and all mycobacteria core genes. *M. immunogenum* species-specific genes, i.e. genes present in *M. immunogenum* but not in other mycobacteria, have been labelled. (d) Venn diagram showing the gene distribution between the two subspecies of *M. immunogenum *. In total, 902 gene clusters are found to be specific to Subspecies 1 and 688 gene clusters to be specific to Subspecies 2.

To investigate the genomic structure and gene content of *M. immunogenum *, we performed pan-genome analysis using the protein sequences of seven *M. immunogenum* strains. All the genome sequences of *M. immunogenum* were annotated using the RAST pipeline to ensure uniformity. Our analysis showed that *M. immunogenum* has an open pan-genome based on the expression $A_{1}∙x^{B_{1}}$ → ∞ as more genomes were added (Fig. 3a). This meant that *y* would increase indefinitely, resulting in an open pan-genome (Fig. 3). For instance, if we add more genomes, this would continue to yield new gene clusters, indicating a high diversity of *M. immunogenum *.

In total, *M. immunogenum* had 4521 core genes within its pan-genome, which consisted of 5562 genes. Interestingly, we noted two distinct patterns of data points for both the pan- and core genome plots. One possible explanation for this is that CD116 might differ from the genomes of Subspecies 1 as it was considered to represent a different subspecies. Thus, we performed another pan-genome analysis (including Subspecies 1 only) by excluding the CD116 genome and found that the variance in the data points for both the pan-genome and core genome become smaller compared to those observed in Fig. 3b and S1. This indicated that the two patterns observed in the original plots are probably influenced by the different subspecies in this species. Together, our data suggest that Subspecies 1 has an open pan-genome.

Comparing the core genes of *M. immunogenum* with the genes from the other mycobacteria revealed 422 genes specific to *M. immunogenum* (Fig. 3c). By manual inspection in the genome browser, a group of *M. immunogenum *-specific genes arranged adjacent to each other into two genomic regions (Table S2) were identified. The close arrangement of these genes may indicate that they could be co-expressed or co-regulated and have similar function. Interestingly, these regions are not found in the other mycobacterial genomes, suggesting that they may contribute to the unique functions of *M. immunogenum *.

The first genomic region has genes involved in the oligopeptide transport system. Oligopeptide transport participates in nutrient uptake and is involved in pathogenesis [45]. For example, the human pathogen *Moraxella catarrhalis* has an oligopetide permease ABC transport operon (*opp*), which maintains bacterial growth in minimal media and mouse respiratory tracts [46]. The operon takes up peptides, which can be digested to produce amino acids necessary for protein synthesis in bacteria. Substrate specificity is determined by *oppA*, whereas the *oppB* protein forms the translocation pore and the *oppF* binds nucleotide [45]. Other members of *opp* have been shown to contribute to modulation of the macrophage immune response by *Mycobacterium tuberculosis* [45]. Other genes in the first genomic region were genes coding for enzymes (e.g. aminopeptidase, *N*-acylamino acid racemase and l-lysine dehydrogenase) that are involved in amino acid metabolism and peptide uptake and digestion.

The second genomic region harboured the non-ribosomal peptide synthetase (NRPS) involved in siderophore biosynthesis. The NRPS involved in the synthesis of peptides for siderophore formation [47] was not found in the genomes of other mycobacterial species, and probably represents a novel feature of *M. immunogenum *. The NRPS allows the synthesis of peptides without the need for a ribosome. In this case, the synthesis of siderophore appeared to be modulated by *lysR* and *luxR* as they occurred in the same cluster. Both of these have been implicated in quorum sensing [48, 49]. Because quorum sensing integrates information from the bacterial environment in order to alter gene expression, we cannot rule out the possibility that both *lysR* and *luxR* could be utilized by *M. immunogenum* in low iron environments (such as in host macrophages) in order to increase siderophore synthesis.

We further investigated the genetic difference between the *M. immunogenum* subspecies by comparing genes/proteins of all strains (Fig. 3d). There were 902 gene clusters specific to Subspecies 1, whereas 688 gene clusters were specific to Subspecies 2. These subspecies-specific genes could be used as biomarkers to differentiate the two subspecies in future.

### GIs and prophage analyses

GIs are clusters of genes in prokaryotic genomes of probable horizontal origin and are often associated with microbial adaptations of medical or environmental interest [50]. Using the IslandViewer software [51], we predicted 74 putative GIs in the genomes of the Subspecies 1 strains. Out of the 74 GIs, nine GIs were present in all the genomes of Subspecies 1. We also found 23 putative GIs in the genome of Subspecies 2. Strikingly, Subspecies 1 has almost three times as many GIs compared to Subspecies 2. The cumulative size of the GIs for Subspecies 1 was much larger compared to that ffor Subspecies 2 (Table S3). Moreover, all of these 23 GIs were not found in the genomes of Subspecies 1, suggesting that they might have been acquired by Subspecies 2 after the divergence of the two subspecies.

**Fig. 4.**
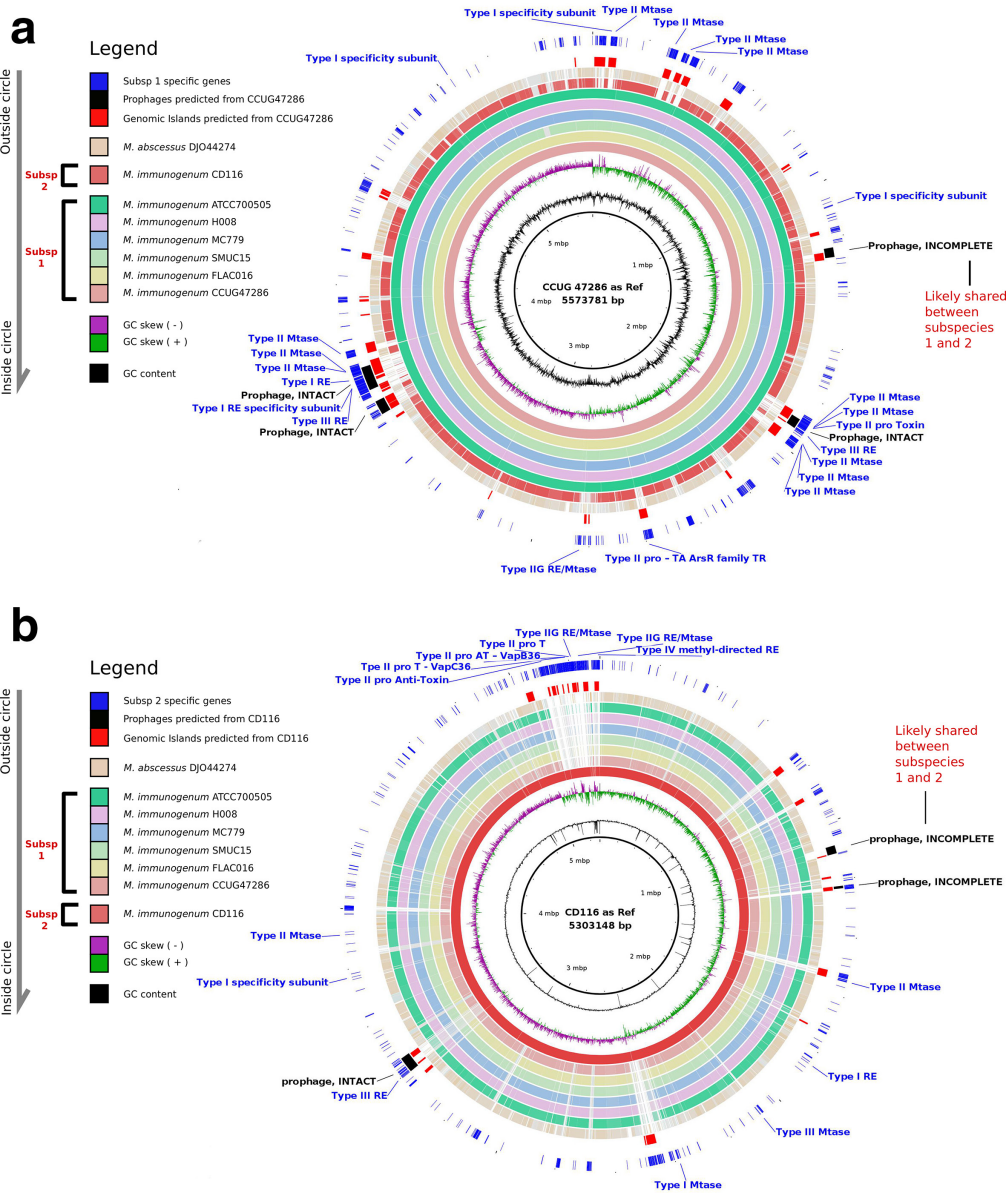
(a) Genome comparison between all *M. immunogenum* strains and *M. abscessus *. (a) *M. immunogenum* CCUG47286 as a reference that represented Subspecies 1. Opaque, coloured regions indicate that the genome is between 90 and 100 % similar to CCUG47286. Semi-transparent regions represent a similarity of 80–90 %. Completely transparent regions represent a similarity <80 %. Unique genes, predicted prophage and GI regions from CCUG47286 are represented in the corresponding outermost three circles. Completion status of prophages and bacterial defence genes found to be unique to Subspecies 1 are labelled on the outermost circle. (b) *M. immunogenum* CD116 as a reference that represented Subspecies 2. Opaque, coloured regions indicate that the genome is between 90 and 100 % similar to CD116. Semi-transparent regions represent a similarity of 80–90 %. Completely transparent regions represent a similarity <80 %. Unique genes, predicted prophage and GI regions from CD116 are represented in the three outermost circles. Completion status of prophage and bacterial defence genes found to be unique to Subspecies 1 are labelled on the outermost circle. Intact prophages probably representing recent phage infection and incomplete prophages probably representing older phage infections are also shown. Prophages probably shared between Subspecies 1 and 2 are also labelled.

Furthermore, we wondered whether the two subspecies of *M. immunogenum* have the same distribution of horizontally transferred prophages. We identified three putative intact prophages in Subspecies 1 and one intact prophage in Subspecies 2 (Table S4). All of these prophages were also GIs. Subspecies 2 appeared to have only an intact prophage, suggesting that it might have been acquired recently. All members of Subspecies 1 had three intact prophages, but all were absent from Subspecies 2, suggesting that they might have been acquired independently by both subspecies. Subspecies 2 had two incomplete (cryptic) prophages, whereas Subspecies 1 had 1.67 incomplete prophages on average. One of the incomplete prophages was found to be common between subspecies 1 and 2, suggesting that they might have been acquired from the common ancestor of the two species (Fig. 4). Details of the prophage distribution are given in Table S3.

**Fig. 5.**
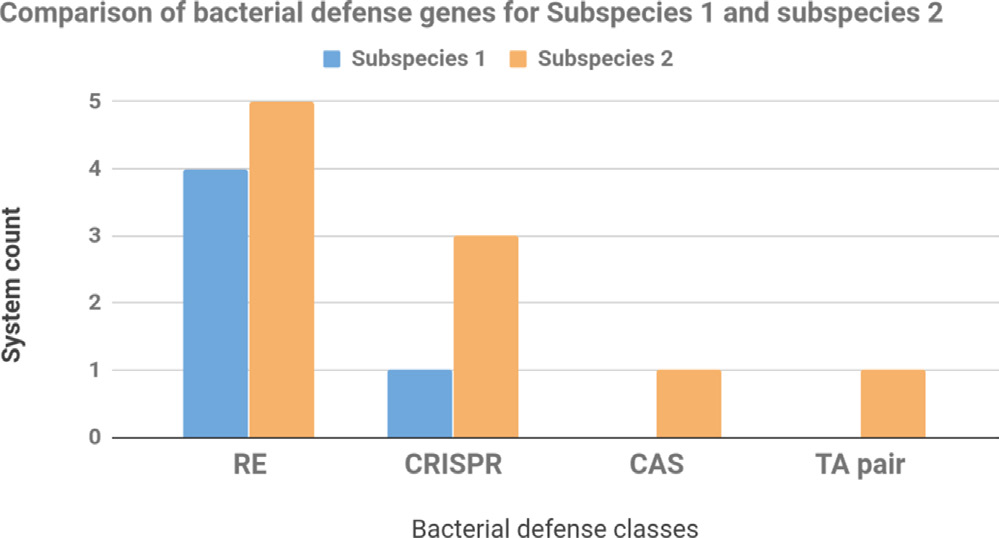
Visual comparison of complete bacterial defence system counts between Subspecies 1 and Subspecies 2 of *M. immunogenum *.

One of the possible explanations for the considerably large difference between the number of GIs in the two subspecies is that there could be some differences in their bacterial defence system. Therefore, we further investigated bacterial defence genes of the two subspecies (Fig. 5). We found that Subspecies 1 had generally fewer genes involved in bacterial defence compared to Subspecies 2. Subspecies 2 had three CRISPR-related genes, whereas Species 1 had only one. Interestingly, Subspecies 2 had genes involved in the CAS and TA systems, whereas no genes were detectable in the genomes of Species 1 for both the CAS and TA systems. Notably, the Cas gene is the component that utilizes the RNA encoded by CRISPR arrays to find and cut bacterial DNA. The CRISPR-Cas system is a defence mechanism for bacteria to develop immunity to phages and plasmids. The presence of these defence genes, especially the Cas genes in Subspecies 2, may partially explain why this subspecies has fewer horizontally transferred GIs and prophages. Subspecies 2 might have a greater ability to defend against the insertion of foreign DNA into its genome.

### dN/dS value distribution indicates relaxed selection pressure

**Fig. 6.**
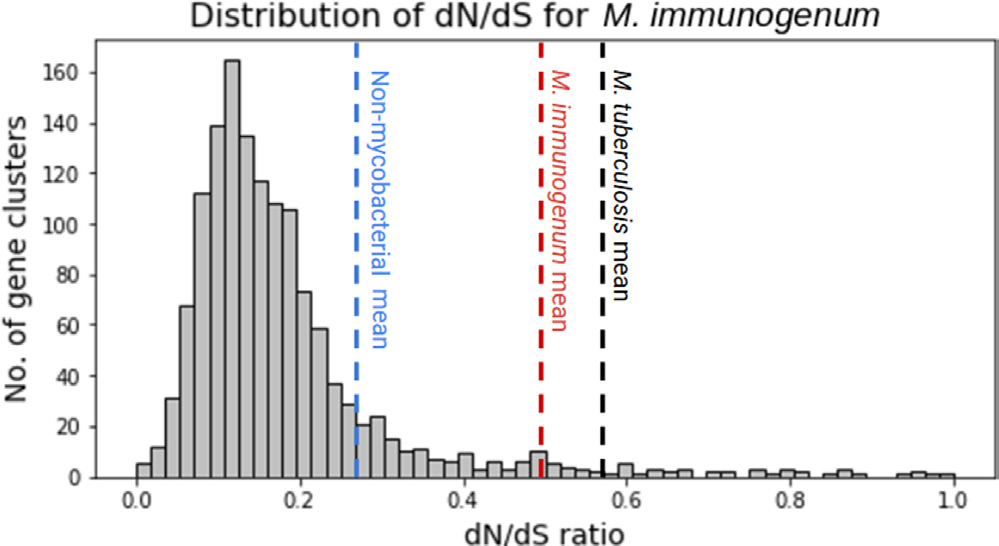
Distribution of dN/dS values for 1:1 orthologues for seven strains of *M. immunogenum *. Blue, red and black dashed lines represent the average dN/dS values for non-mycobacterial bacteria, *M. immunogenum* and *Mycobacterium tuberculosis *, respectively.

To further investigate the evolution of the emerging pathogen *M. immunogenum *, we performed positive selection analysis. The distribution of dN/dS values for 1:1 orthologues for *M. immunogenum* was found to be positively skewed with a median of 0.16 and a mean of 0.49 (Fig. 6). Mean dN/dS values of non-pathogenic mycobacteria and the pathogenic *Mycobacterium tuberculosis* were also plotted for comparisons. *Mycobacterium tuberculosis* had the highest dN/dS values, followed by *M. immunogenum* and then the non-mycobacterial bacteria.

Our results showed that the pathogenic *M. immunogenum* consists of 13 positively selected genes compared to non-pathogenic mycobacteria (Table 1). The positively selected genes may contribute to the survival of *M. immunogenum *. Our data showed that many proteins play an important role in virulence. For example, the iron binding ABC transporter enables pathogenic bacteria to survive inside host macrophages depleted in iron in order to kill bacteria [52]. NADPH:quinone dehydrogenase detoxifies quinone [53], which is an antibacterial compound. The fatty acid desaturase has been demonstrated to be a requirement for virulence [54]. Aldehyde dehydrogenases protect against nitrosative stress in *Mycobacterium tuberculosis* [55]. Uridylyltransferase is involved in the synthesis of glycolipids, glycoproteins and proteoglycans for the formation of cell walls [56]. Interestingly, the structure of uridylyltransferase is different for prokaryotes than for eukaryotes [57], and thus antibiotics targeting it could be developed while also leaving the eukaryotic version of the protein unharmed. The transcription elongation factor *greA* has been linked to heat shock and oxidative stress resistance [58]. Other proteins are either hypothetical or have functions which were undetermined, and therefore we cannot rule out the possibility that they could also contribute to virulence.

**Table 1.** List of genes under positive selection LnL refers to the Log likelihood value. LRT is the Likelihood Ratio Test score, as compared between the null and alternative hypothesis. Multiple correction was performed using the Benjamini–Hochberg method with a false discovery rate of 0.25. Fore and Back Ω represent *M. immunogenum* and non-pathogenic mycobacteria dN/dS values respectively. Positions positively selected refers to the amino acid position number and code on the translated protein found using Bayesian Empirical Bayes analysis.

Peg id	LnL	Fore Ω	Back Ω	LRT	Start	Stop	Strand	Function	Positions positively selected
534	−7083.38	5.55	0.09	11.63	517 897	518 322	+	Hypothetical protein	148P*, 149A*, 150T, 381A
550	−4461.04	8.79	0.14	13.84	533 773	534 516	+	Hypothetical protein	97K*, 117A, 184D
685	−4279.79	5.65	0.11	20.18	663 075	662 083	−	ABC transporter, substrate-binding protein (cluster 8, B12/iron complex)	242P**, 316S*
894	−3835.12	11.92	0.14	22.2	866 477	867 403	+	NADPH:quinone reductase or related Zn-dependent oxidoreductase	4P, 7T**, 8A
898	−9307.6	0.86	0.15	11.13	872 847	871 840	−	Fatty acid desaturase occurring in virulence cluster	62S, 493Q
932	−5721.86	1.43	0.13	10.5	911 758	913 224	+	Aldehyde dehydrogenase (EC 1.2.1.3)	132D, 194A, 384S**
1091	−5337.66	12.24	0.21	10.28	1 089 456	1089 304	−	cocE/nonD family hydrolase [* Duganella* sp. CF517]	176L, 217L, 280A**, 397L, 407I, 422V
1096	−6060.92	2.43	0.13	9.61	1 092 481	1 092 678	+	Hypothetical protein	388T*
1165	−4011.09	8.74	0.1	9.42	1 159 704	1 159 030	−	Heat shock protein 22.5 (hsp22.5)	32A, 210T, 264K
1168	−3301.42	13.87	0.23	14.91	1 160 826	1 161 755	+	UTP-glucose-1-phosphate uridylyltransferase (EC 2.7.7.9)	52L, 66L, 102T
1323	−2374.38	34.09	0.15	14.73	1 310 113	1 309 619	−	Transcription elongation factor greA	2A, 5Q**, 11A, 14E, 27M
1619	−3188	57.7	0.07	72.99	1 612 933	1 613 244	+	ATP/GTP-binding protein	220E **, 222R*, 223K**, 224L**, 225M*, 228T**, 229Y, 230V**, 232I*, 235M*, 236T, 238F*, 239Q**
1774	−10641.11	1.23	0.12	19.2	1 776 879	1 778 309	+	Carbon monoxide oxidation accessory protein coxE	708D**

Asterisks a probability value greater than *0.95 and greater than **0.99.

## Discussion

Here we performed a comparative analysis of *M. immunogenum* genomes using bioinformatics approaches. We identified the taxonomic position of *M. immunogenum* using approaches similar to Gupta *et al*. [1], such as 16S rRNA, multilocus sequence analysis, core genome SNP, ANI and GGDC methods. Consistent with their findings, our phylogenetic analysis showed that *M. immunogenum* was classified as an RGM and ts closest relatives are *M. abscessus* and *M. chelonae *. Our within-species phylogenetic analyses have for the first time revealed two distinct clusters within *M. immunogenum *. Further analyses showed that they might represent two potential subspecies of *M. immunogenum* supported by the evidence from *in silico* DDH and GGDC analyses. Our data showed that *M. immunogenum* has an open pan-genome, suggesting this bacterium may continue to acquire new genes from other sources. Our positive selection analysis identified *M. immunogenum* genes that have undergone significant positive selection compared to non-pathogenic mycobacteria. Many of these positively selected genes are involved in virulence, suggesting that the mutations might play important roles in transforming the environmental bacterium to a pathogenic bacterium over evolutionary time.

Furthermore, we found a large number of putative horizontally transferred GIs in *M. immunogenum* genomes, suggesting that horizontal gene transfer may play important role in the evolution of this bacterium. Strikingly, the number of GIs of the members of Subspecies 1 is three times the number of GIs found in Subspecies 2. One of the possible explanations is that the Subspecies 2 strain might have lost the GIs over evolutionary time. Another possibility is that Subspecies 2 might have acquired the GIs from other sources at a slower rate compared to the members of Subspecies 1. We believe that the latter is more likely because Subspecies 2 is well equipped with many genes involved in bacterial defence, including the CRISPR-Cas system, allowing Subspecies 2 to prevent insertion of foreign DNA and phages into its genome.

Our data showed that the majority of GIs did not appear to be shared between the two subspecies of *M. immunogenum *, and therefore their divergence might occur before the GI insertions. The lack of shared GIs could be linked to different geographical isolation, as the members of Subspecies 1 were isolated from the USA and France, whereas Subspecies 2 was isolated from India (Fig. S2). It is likely that these GI regions were a primary contributor to the divergence of these subspecies. These regions were not shared between the subspecies, suggesting that they were not transferring these GIs between each other, thus negating distributive conjugal transfer between subspecies. This also indicates that the recent prophage infections, represented by the intact prophages, probably occurred after the subspecies divergence.

Our study showed that Subspecies 2 may be more conserved than Subspecies 1, supported by the presence of important defence genes and the lower number of GIs and prophages. Its conserved nature can be further supported by the large variance seen in the pan-genome plot (Fig. 3), which suggested that the two subspecies may have different pan-genome profiles. For example, Subspecies 1 has an open pan-genome, whereas Subspecies 2 may have a closed pan-genome. However, a limitation of this study is that we only have one strain for Subspecies 2, and therefore we cannot perform pan-genome analysis for Subspecies 2 alone. We cannot rule out the possibility that Subpescies 2 is strain-specific rather than Subspecies 2-specific. It would be of value to isolate and sequence more genomes of Subspecies 2 strains in order to further validate our findings.

## Conclusion

This study reveals for first time the possibility that the pathogenic *M. immunogenum* has two potential subspecies. Both horizontal gene transfer and mutations might have played important roles in the diversification and evolution of this pathogenic species. This comparative genome study provides novel insights into the genome structure and contents of *M. immunogenum* and lays the foundation for future functional work on this pathogen.

### Data accessibility

All data are available in the main text and supporting information.

## Supplementary Data

Supplementary material 1
