# Supplementary material for: Comparative genome analyses of Mycobacteroides immunogenum reveals two potential novel subspecies
Source: Microb Genom. 2020 Dec 9;6(12):mgen000495. doi: 10.1099/mgen.0.000495 (PMC8116688; doi:10.1099/mgen.0.000495)
Supplement: Supplementary material 1 [file mgen-6-495-s001.pdf]

Supplementary Table 1: Genomic and phenotypic properties of 31 mycobacteria used in analysis. (Env. is the abbreviated form of "Environmental")

Species	Strain name	Growth rate	Mode of life	Source	Genomic size (Mbp)	GC (%)	Contig No.	CDS	Assembly accession no.	Genome status
M. immunogenum	CCUG47286	Rapid	Env. pathogen	Bronchoscope washer	5.57	64.30	1	5559	GCA_001605725.1	Complete
	FLAC016	Rapid	Env. pathogen	Human	5.60	64.30	1	5594	GCA_001677135.1	Complete
	ATCC700505	Rapid	Env. pathogen	Metalworking fluid	5.54	64.30	35	5554	GCA_002101665.1	Draft
	H008	Rapid	Env. pathogen	Drinking water	5.72	64.00	46	5850	GCA_001296275.1	Draft
	MC779	Rapid	Env. pathogen	Bronchoalveolar fluid	5.54	64.30	22	5552	GCA_002013555.1	Draft
	SMUC14	Rapid	Env. pathogen	Brain abscess	5.57	64.30	104	5634	GCA_000878425.1	Draft
	CD116	Rapid	Env. pathogen	Duodenal mucosa of celiac disease patient	5.27	64.40	35	5199	GCA_001655155.1	Draft
M. africanum	25	Slow	Obligate pathogen	Human sputum	4.39	65.60	1	5114	GCA_001544855.1	Complete
M. avium	RCAD0278	Slow	Env. pathogen	Farm duck	4.95	69.30	1	4719	GCA_001683455.1	Complete
M. bovis	BCG1	Slow	Obligate pathogen	Cow seed lot	4.36	65.60	38	4270	GCA_001293105.1	Draft
M. colombiense	CECT3035	Slow	Env. pathogen	Human blood	5.58	68.10	1	5453	GCA_002105755.1	Complete
M. intracellulare	1956	Slow	Env. pathogen	Human sputum	5.49	68.10	3	4930	GCA_000523815.1	Draft
M. kansasii	ATCC12478	Slow	Env. pathogen	Human, fatal case	6.58	66.20	1	5970	GCA_000157895.2	Complete
M. leprae	TN	Slow	Env. pathogen	Armadillo	3.27	57.80	1	6218	GCA_000195855.1	Complete
M. marinum	1218R	Slow	Env. pathogen	Fish	6.30	65.30	509	5753	GCA_002911595.1	Draft
M. microti	12	Slow	Obligate pathogen	Human	4.37	65.60	1	4585	GCA_001544815.1	Complete

Species	Strain name	Growth rate	Mode of life	Source	Size (Mbp)	GC %	Contig no.	CDS	Assembly accession no.	Genome status
M. parascrofulaceum	ATCCBAA614	Slow	Env. pathogen	Human vaginal urogenital tract	6.56	67.60	405	6391	GCA_000164135.1	Draft
M. simiae	MO323	Slow	Env. pathogen	Human bronchial lavage	5.94	66.30	1	5774	GCA_001584765.1	Complete
M. tuberculosis	H37RV	Slow	Obligate pathogen	Human	4.41	65.60	1	4299	GCA_000195955.2	Complete
M. ulcerans	AGY99	Slow	Env. pathogen	Human tissue biopsy	5.81	65.40	1	5630	GCA_000013925.2	Complete
M. abscessus	DJO44274	Rapid	Env. pathogen	Human fluid	4.69	64.12	1	4640	GCF_000770175.1	Complete
M. chelonae	CCUG47445	Rapid	Env. pathogen	Tortoise tubercle	5.03	63.90	1	4957	GCA_001632805.1	Complete
M. chubuense	NBB4	Rapid	Env. non-pathogen	Creosote-contaminated soil	6.34	68.30	1	5325	GCA_000266905.1	Complete
M. fortuitum	CT6	Rapid	Env. pathogen	Rhizosphere soil	6.25	66.20	1	6064	GCA_001307545.1	Complete
M. gilvum	SPYR1	Rapid	Env. non-pathogen	Creosote contaminated soil	5.78	67.80	1	5355	GCA_000184435.1	Complete
M. hassiacum	DSM44199	Rapid	Env. non-pathogen	Human urine	5.08	69.40	104	4963	GCA_000379865.1	Draft
M. mageritense	DSM44476	Rapid	Env. pathogen	Sputum from asymptomatic human	7.97	66.90	6	7795	GCA_000612825.1	Draft
M. phlei	CCUG21000	Rapid	Env. pathogen	Unknown	5.35	69.40	1	5180	GCA_001583415.1	Complete
M. smegmatis	INHR1	Rapid	Env. non-pathogen	Human smegma	6.99	67.40	1	6844	GCA_000767665.1	Complete
M. vaccae	95051	Rapid	Env. non-pathogen	Cow milk	6.24	68.60	1	5959	GCA_001655245.1	Complete
M. vanbaalenii	PYR1	Rapid	Env. non-pathogen	Petrogenic chemical contaminated water	6.49	67.80	1	6268	GCA_000015305.1	Complete

Supplementary Table 2. Genomic regions with novel genes found in *M. immunogenum* specific genes. Consecutive protein encoding gene IDs indicate that these genes are adjacent to each other. The start and stop positions refer to the locations of the genes in the genome of *M. immunogenum* CCUG47286. Genes which could not be found in the other mycobacteria through a BLASTP search were bolded and have an asterisk next to their Peg ID.

Genomic region	Peg ID	Start	Stop	Translated product
1	4344	4303756	4302188	N-acyl-D-amino-acid deacylase (EC 3.5.1.81)
	**4345 ***	**4306337**	**4303749**	**Membrane alanine aminopeptidase N (EC 3.4.11.2)**
	4346	4307927	4306392	hypothetical protein
	4347	4308812	4307976	Oligopeptide transport ATP-binding protein OppF (TC 3.A.1.5.1)
	**4348 ***	**4309813**	**4308809**	**Oligopeptide transport system permease protein OppB (TC 3.A.1.5.1)**
	4349	4310724	4309810	hypothetical protein
	4350	4311788	4310721	ABC transporter, permease protein 1 (cluster 5, nickel/peptides/opines)
	4351	4313692	4311785	Oligopeptide ABC transporter, periplasmic oligopeptide-binding protein OppA (TC 3.A.1.5.1)
	4353	4316794	4315166	PucR transcriptional regulator
	**4354 ***	**4316873**	**4317805**	**acetyltransferase**
	**4355 ***	**4317802**	**4318923**	**N-acylamino acid racemase**
	4356	4318965	4320050	L-lysine dehydrogenase
2	2760	2779572	2778967	NADH oxidoreductase
	2761	2779650	2780534	Transcriptional regulator, LysR family
	2762	2781219	2780587	Two-component transcriptional response regulator, LuxR family
	2763	2782337	2781216	hypothetical protein
	**2764 ***	**2782507**	**2785599**	**Siderophore biosynthesis non-ribosomal peptide synthetase modules**
	2765	2785596	2793068	hypothetical protein
	2766	2793065	2793970	hypothetical protein

Supplementary Table 3: The GI distribution of seven *M. immuogenum* strains.

GIs	Length(bp)	ATCC700505	CD116	H008	MC779	SMUC14	CCUG47286	FLAC016
GI15	33,822		\					
GI30	26,335		\					
GI31	25,468		\					
GI32	23,459		\					
GI37	18,556		\					
GI38	18,091		\					
GI39	16,248		\					
GI42	14,762		\					
GI43	14,742		\					
GI45	13,686		\					
GI46	13,507		\					
GI50	11,123		\					
GI65	7,165		\					
GI70	6,603		\					
GI74	5,797		\					
GI75	5,753		\					
GI76	5,729		\					
GI79	5,048		\					
GI86	4,786		\					
GI87	4,715		\					
GI89	4,506		\					
GI93	4,403		\					
GI94	4,358		\					
GI1	59,760	\		\	\		\	\
GI2	58,432	\		\	\	\	\	\
GI3	54,839	\			\	\	\	\
GI4	54,741					\		
GI5	53,275	\		\	\	\	\	\
GI6	19,176	\		\	\	\	\	\
GI7	42,909					\		
GI8	42,559					\		
GI9	40,862	\						
GI10	40,176	\		\	\	\	\	\
GI11	39,888	\			\	\	\	\
GI12	3,979						\	
GI13	39,080				\			\
GI14	38,836						\	
GI16	33,161	\		\	\	\	\	\
GI17	30,973				\			
GI18	30,352						\	\
GI19	29,739	\		\	\		\	\
GI20	28,117	\		\	\	\	\	\
GI21	28,950					\		
GI22	28,539						\	\
GI23	27,914					\		
GI24	27,387						\	\
GI25	27,309				\			
GI26	27,298	\						
GI27	26,797			\				
GI28	26,403							\
GI29	26,403						\	
GI33	23,404	\		\	\	\	\	\
GI34	22,350						\	\

GI35	22,896			\				
GI36	16,908	\			\	\	\	\
GI40	16,210						\	\
GI41	15,608							\
GI44	13,767			\				
GI47	12,532	\		\	\	\	\	\
GI48	11,970			\				
GI49	11,592	\		\	\	\	\	
GI51	11,088	\			\	\	\	\
GI52	10,588							\
GI53	10,508					\		\
GI54	10,394	\			\	\	\	\
GI55	10,197			\				
GI56	9,937	\			\		\	
GI57	9,062	\		\	\	\	\	\
GI58	9,046							\
GI59	8,766						\	\
GI60	8,555						\	\
GI61	8,527						\	\
GI62	8,306						\	\
GI63	7,915							\
GI64	7,666			\		\		\
GI66	7,090						\	\
GI67	7,020			\		\		\
GI68	6,811							\
GI69	6,804						\	\
GI71	6,499						\	\
GI72	6,457			\				
GI73	6,281	\			\			
GI77	5,491					\		
GI78	5,210						\	\
GI80	5,009	\				\		
GI81	4,975						\	\
GI82	4,960						\	\
GI83	4,863						\	\
GI84	4,854						\	\
GI85	4,819	\			\	\	\	\
GI88	4,517							\
GI90	4,466						\	\
GI91	4,453						\	\
GI92	4,447			\				
GI95	4,293	\			\	\	\	\
GI96	4,035					\		
GI97	3,674	\						

Supplementary Table 4: The predicted prophages in both subspecies. Total number of prophages of each strain were shown.

	Strain	Intact	Questionable	Incomplete	Total
Subspecies 1	ATCC700505	3	1	1	5
	CCUG47286	3	0	1	4
	FLAC016	3	1	0	4
	H008	3	2	5	10
	MC779	3	1	1	5
	SMUC14	3	0	2	5
Subspecies 2	CD116	1	0	2	3

[a] The completeness score can be interpreted as an indicator of whether a prophage was complete or if it was defective/cryptic. If the completeness score was above 90, it was intact, which means it had the gene repertoire necessary to create new phage particles and infect other bacteria. If completeness score was between 60 and 90, it was considered questionable while below 60 was considered incomplete. They were considered indicators of defective/cryptic prophage(43).

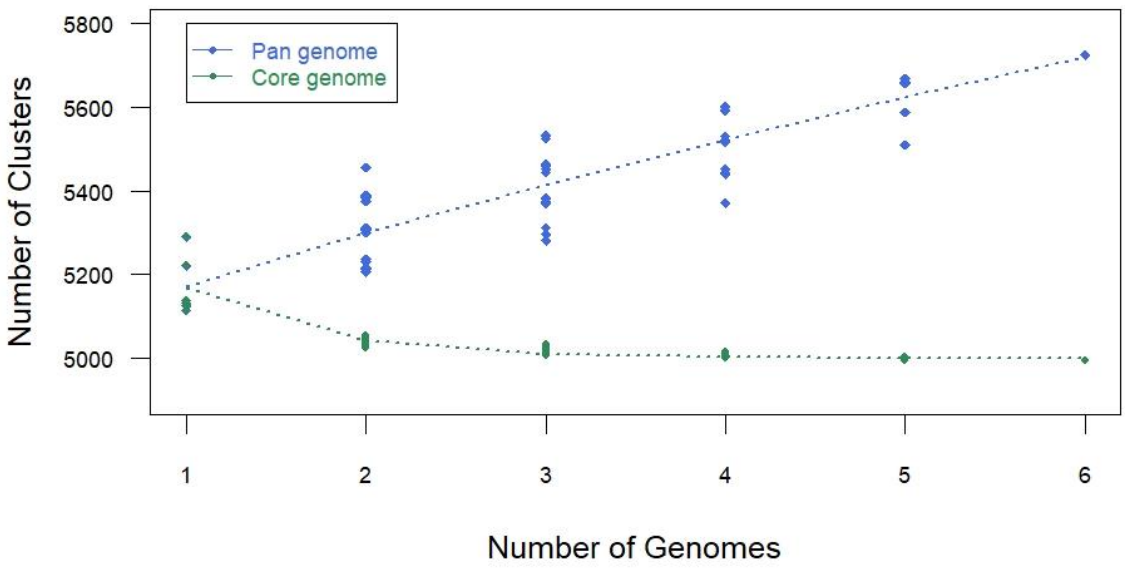

Supplementary Figure 1. Pan-genome analysis of *M. immunogenum* Subspecies 1. Plot of number of gene clusters in pan genome and core genome vs. number of genomes sampled for six *M. immunogenum* Subspecies 1 genomes. Blue data points indicate the number of pan genome clusters (total number of clusters) belonging to the sampled number of genomes. Likewise, the green data points indicate the number of core genome clusters, i.e. gene clusters present in the sampled number of genomes.

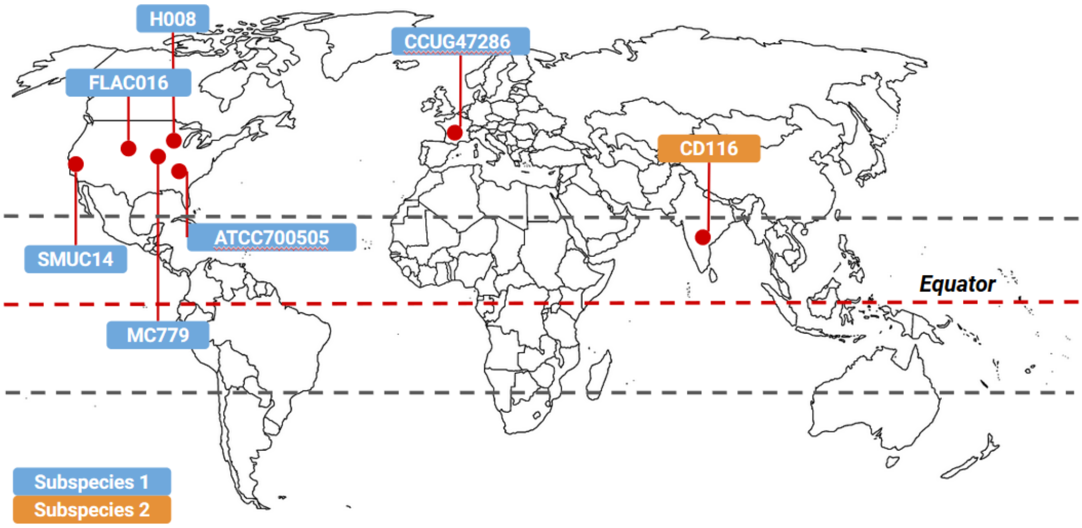

Supplementary Figure 2. Geographical isolations of *M. immunogenum* strains. The three dashed lines, starting from the top represent the tropic of cancer, the equator and the tropic of Capricorn, respectively. Subspecies 1 members were originated from the temperate regions above the tropic of cancer, whereas the Subspecies 2 strain was originated from the equator.
